# Understanding the cluster randomised crossover design: a graphical illustraton of the components of variation and a sample size tutorial

**DOI:** 10.1186/s13063-017-2113-2

**Published:** 2017-08-15

**Authors:** Sarah J. Arnup, Joanne E. McKenzie, Karla Hemming, David Pilcher, Andrew B. Forbes

**Affiliations:** 1 School of Public Health and Preventive Medicine, Monash University, The Alfred Centre, Melbourne, VIC 3004 Australia; 2 Institute of Applied Health Research, University of Birmingham, Edgbaston, Birmingham, B15 2TT UK; 3 Australian and New Zealand Intensive Care Society Centre for Outcome and Resource Evaluation, Ievers Terrace, Carlton, VIC 3154 Australia; 4 Department of Intensive Care, The Alfred Hospital, Commercial Road, Melbourne, VIC 3004 Australia; 5 Australian and New Zealand Intensive Care Research Centre, School of Public Health and Preventive Medicine, Monash University, The Alfred Centre, Melbourne, VIC 3004 Australia

**Keywords:** Cluster randomised, Crossover, Sample size, Intracluster correlation, Within-period correlation, Between-period correlation, Components of variability

## Abstract

**Background:**

In a cluster randomised crossover (CRXO) design, a sequence of interventions is assigned to a group, or ‘cluster’ of individuals. Each cluster receives each intervention in a separate period of time, forming ‘cluster-periods’. Sample size calculations for CRXO trials need to account for both the cluster randomisation and crossover aspects of the design. Formulae are available for the two-period, two-intervention, cross-sectional CRXO design, however implementation of these formulae is known to be suboptimal. The aims of this tutorial are to illustrate the intuition behind the design; and provide guidance on performing sample size calculations.

**Methods:**

Graphical illustrations are used to describe the effect of the cluster randomisation and crossover aspects of the design on the correlation between individual responses in a CRXO trial. Sample size calculations for binary and continuous outcomes are illustrated using parameters estimated from the Australia and New Zealand Intensive Care Society – Adult Patient Database (ANZICS-APD) for patient mortality and length(s) of stay (LOS).

**Results:**

The similarity between individual responses in a CRXO trial can be understood in terms of three components of variation: variation in cluster mean response; variation in the cluster-period mean response; and variation between individual responses within a cluster-period; or equivalently in terms of the correlation between individual responses in the same cluster-period (within-cluster within-period correlation, WPC), and between individual responses in the same cluster, but in different periods (within-cluster between-period correlation, BPC).

The BPC lies between zero and the WPC. When the WPC and BPC are equal the precision gained by crossover aspect of the CRXO design equals the precision lost by cluster randomisation. When the BPC is zero there is no advantage in a CRXO over a parallel-group cluster randomised trial. Sample size calculations illustrate that small changes in the specification of the WPC or BPC can increase the required number of clusters.

**Conclusions:**

By illustrating how the parameters required for sample size calculations arise from the CRXO design and by providing guidance on both how to choose values for the parameters and perform the sample size calculations, the implementation of the sample size formulae for CRXO trials may improve.

**Electronic supplementary material:**

The online version of this article (doi:10.1186/s13063-017-2113-2) contains supplementary material, which is available to authorized users.

## Background

Individually randomised trials are considered the ‘gold standard’ for evaluating medical interventions [1]. However, situations arise where is it necessary, or preferable, to randomise clusters of individuals, such as hospitals or schools, rather than the individual patients or students, to interventions [2, 3]. A cluster randomised trial will generally require a larger sample size compared with an individually randomised trial to estimate the intervention effect to the same precision [4].

In a two-period, two-intervention, cluster randomised crossover (CRXO) design, each cluster receives each of the two interventions in a separate period of time, leading to the formation of two ‘cluster-periods’. In a cross-sectional design, each cluster-period consists of different individuals, while in a cohort design, each cluster-period consists of the same individuals. The order in which the interventions are delivered to each cluster is randomised to control for potential period effects [5, 6]. Like in an individually randomised trial, this adaption has the benefit of reducing the required number of participants [7]. The key to understanding the CRXO design is to recognise how both the cluster randomisation and crossover aspects of the design lead to variation between individual responses in a trial; and how these aspects of the design give rise to similarities in the responses of groups of individuals.

Sample size formula have been published for the two-period, two-intervention, cross-sectional CRXO design [8–10]. These formulae require a-priori specification of two correlations: the similarity between two individuals in the same cluster-period, typically measured by the within-cluster within-period correlation (WPC); and the similarity between two individuals in the same cluster, but in different cluster-periods, typically measured by the within-cluster between-period correlation (BPC). However, there is little guidance for informing the value of the BPC, nor on the sensitivity of the sample size to the chosen values of both correlations [11, 12].

A 2015 systematic review of CRXO trials found that both the cluster randomisation and crossover aspects of the design of the CRXO was appropriately accounted for in only 10% of sample size calculations and 10% of analyses [13]. This suggests that the CRXO design is not well understood.

The aims of this tutorial are to illustrate the intuition behind the CRXO design; to provide guidance on how to a-priori specify the WPC and BPC; and perform sample size calculations for two-period, two-intervention, cross-sectional CRXO trials.

In the ‘Understanding the CRXO design’ section, we describe how the cluster randomisation and crossover aspects of the design leads to variation between individual responses in a two-period, two-intervention, cross-sectional CRXO design, using intensive care unit (ICU) length(s) of stay (LOS) as an example. In the ‘Performing a sample size calculation’ section, we outline how to perform sample size calculations and discuss how to specify values of the WPC and BPC for sample size calculations. In the ‘Common mistakes when performing a sample size analyses’ section, we outline common mistakes made by trialists when performing sample size calculations for CRXO trials and the likely consequences of those mistakes. We conclude with a general discussion, considering extensions and larger designs.

## Understanding the CRXO design

In this section we illustrate graphically how the cluster randomisation and crossover aspects of the CRXO design leads to variation in the responses of individuals in a CRXO trial, and how these aspects of the design can be used to measure the similarity between individuals using the WPC and BPC.

We illustrate the sources of variation and measures of similarity that arise in the two-period, two-intervention, cross-sectional CRXO design by considering a hypothetical CRXO trial conducted in 20 ICUs over a 2-year period. We consider the ICU LOS of all patients admitted to these 20 ICUs, and assume (for ease of exposition) that the number of patients in each ICU is infinitely large (or at least very large). As LOS is non-normally distributed and right skewed, we use the logarithmic transform of ICU LOS throughout our illustration.

Each ICU is randomly assigned to administer one of two interventions to all patients admitted during the first year (period 1). In the subsequent year, each ICU administers the alternate intervention (period 2). All patients admitted to a single ICU over the 2-year period can be thought of as belonging to a *cluster*. Within each ICU (cluster), the patients admitted during a 1-year period can be thought of as belonging to a separate *cluster-period*. Therefore, in each ICU (cluster) there are two cluster-periods.

**Fig. 1:**
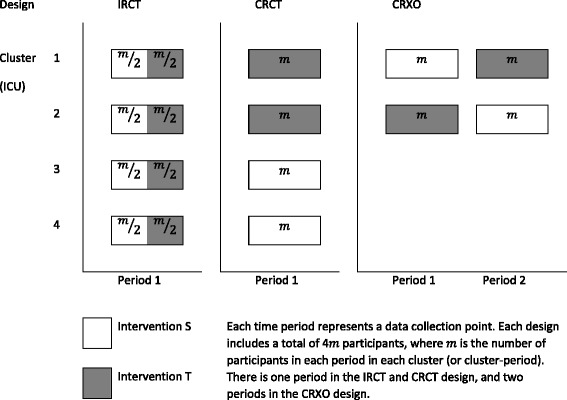
Schematic illustration of the stratified, multicentre, parallel-group, individually randomised trial (IRCT), parallel-group cluster randomised trial (CRCT), and cluster randomised crossover (CRXO) design with the same total number of participants

The allocation of interventions to patients in the stratified, multicentre, parallel-group, individually randomised trial (IRCT) design, the parallel-group cluster randomised trial (CRCT) design, and the CRXO design are shown in Fig. 1. In each design, each intervention is given for one 12-month period. In the IRCT design half the patients in each centre (ICU) receive each intervention. In the CRCT design, all patients in a single ICU are assigned the same intervention.

### Variation in the length of stay between patients

To illustrate the sources of variation and measures of similarity that arise in the CRXO design, we assume that the true difference between interventions is zero. In the hypothetical situation where we have an infinite number of patients, the overall mean LOS for all patients in the trial will be equal to the true overall mean LOS for all patients who could be admitted to the 20 ICUs. The variation in LOS arises from both patient and ICU factors. In a CRXO design, the ICU (cluster) and the time period of admission (cluster-period) are both factors that could affect the patient’s LOS and, therefore, explain some of the variation seen in patient LOS. For example, each ICU may have a different case mix of patients, different operating policies and procedures, and different staff. And within an ICU, changes to staff or policy over time could lead to differences in LOS between time periods. The following sections describe how the ICU and time period of admission can explain part of the variation in the LOS between patients.

### Variation in the length of stay between ICUs

Each ICU has a true mean LOS for the infinite number of patients who could be hypothetically admitted to that ICU. When there is true variability between ICUs, the true mean LOS for each ICU will differ from the mean of all true ICU mean LOS. In the hypothetical situation where we have an infinite number of patients, the overall mean LOS for all patients and the mean of all true ICU mean LOS will be equal to the same true overall mean LOS.

**Fig. 2:**
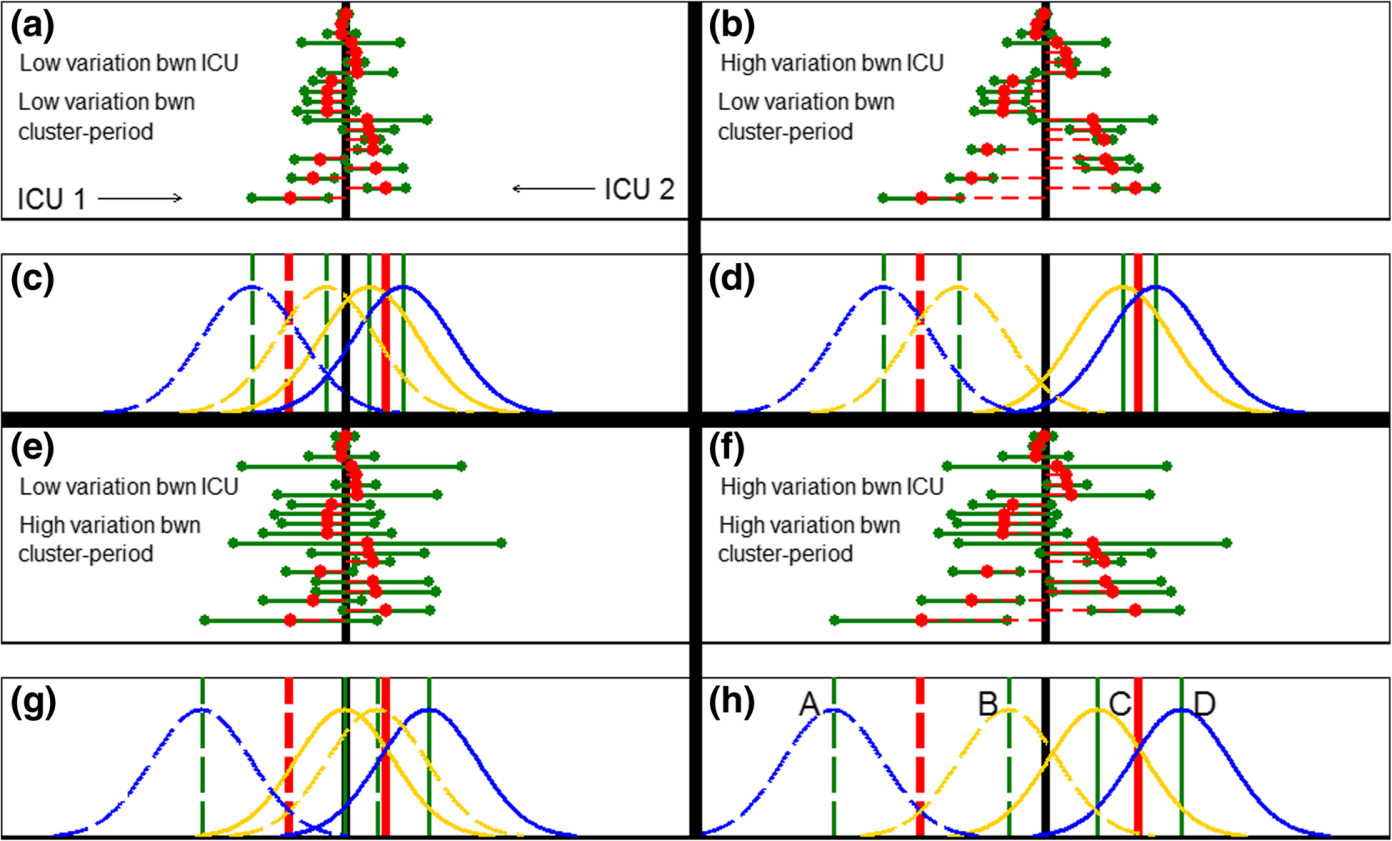
Variation in true mean length(s) of stay (LOS) between intensive care units (ICUs) and between periods within ICUs. Low variation in the true mean LOS between ICUs is shown in the left column (**a**, **c**, **e**, **g**) and high variation in the right column (**b**, **d**, **f**, **h**). Low variation in the true mean LOS between periods within ICUs is shown in the top row (**a**, **c**, **b**, **d**) and high variation in the bottom row (**e**, **g**, **f**, **h**). **a**, **b**, **e**, **f** the true mean LOS for each of the 20 hypothetical ICUs are marked by a *red circle*, with the difference between the true overall mean LOS and the true mean LOS for each ICU indicated by a *dashed red horizontal line*. The two true cluster-period mean LOS for each ICU are marked with a *green circle* to the left and right of the true ICU mean LOS. The difference between the true ICU mean LOS and the true cluster-period mean LOS is indicated by a *green horizontal line*. The *black vertical line* indicates true overall mean LOS. **c**, **d**, **g**, **h** the *red vertical line* indicates the true ICU mean LOS and the *green vertical line* indicates the true cluster-period mean LOS for each period in each of two ICUs. For (**a**) *WPC* = 0.02, *BPC* = 0.01; for (**b**) *WPC* = 0.06, *BPC* = 0.05; for (**e**) *WPC* = 0.06, *BPC* = 0.01; for (**f**) *WPC* = 0.10, *BPC* = 0.05. ICU 1 is shown with *solid lines* and ICU 2 is shown in *dashed lines* in (**h**). The *yellow (blue) curve* indicates a normal distribution of patient LOS within each cluster-period where the cluster was allocated to intervention S (T). For (**d**) the distribution of patient LOS in each of the four cluster-periods are labelled A to D. WPC: within-cluster within-period correlation (*ρ*); BPC: within-cluster between-period correlation (*η*)

Figure 2a, b, e and f show four scenarios that each illustrate variation in the true mean LOS across ICUs (*red circles*). The true mean LOS in each ICU may be similar and, therefore, close to the true overall mean LOS (*black line*) (Fig. 2a); or the true mean LOS of each ICU may be more dispersed about the true overall mean (Fig. 2b). The difference in the spread of true ICU mean LOS between Fig. 2a and b indicates greater variability in the true ICU mean LOS across ICUs in Fig. 2b than in Fig. 2a. The same comparison can be made between Fig. 2e and f.

### Variation in the length of stay between time periods in an ICU

Within each ICU, there is also a true mean LOS for the infinite number of patients who could be hypothetically admitted in each 1-year period (i.e. each cluster-period). Figure 2a, b, e and f show also that there is variation in the difference between the true cluster-period mean LOS (*green circles*) and the true ICU mean LOS (*red circles*). The true cluster-period mean LOS may be similar to the true ICU mean LOS Fig. 2a); or the true mean LOS of each cluster-period may be more dispersed about the true ICU mean (Fig. 2e). The difference in the spread of the true cluster-period mean LOS between Fig. 2a and e indicates greater variability in true cluster-period mean LOS within ICUs in Fig. 2e than in Fig. 2a. The same comparison can be made between Fig. 2b and f.

### Variation in length of stay between patients in a cluster-period

While there is a true mean LOS for all patients admitted in each cluster-period, the individual patients within each cluster-period will show variation in their LOS due to other patient factors (e.g. severity of their condition).

Two of the 20 example ICUs are depicted in Figs. 2c, d, g and h. ICU 1 is shown with *solid lines* and ICU 2 is shown in *dashed lines*. As previously, the mean LOS in each ICU is marked by a *red line*, and the mean LOS in each cluster-period is marked by a *green line*. The distribution of the individual patient LOS within each cluster-period follows a normal distribution, and is shown with four *yellow or blue curves*. The distribution of the LOS for patients receiving intervention S are coloured *yellow*, and the distribution of those receiving intervention T are coloured *blue*.

Within each cluster-period, patients have a range of individual LOS centred at the true cluster-period mean LOS (*green line*). Nonetheless, the patients in each cluster-period are from distinct distributions labelled as A, B, C, and D in Fig. 2h (these labels apply also to Fig. 2c, d and g). In each cluster-period, we assume that the variability of the individual patient LOS is the same, and hence the *yellow and blue curves* have the same shape and are only shifted in location between the four cluster-periods.

### Summary of the sources of variation in the CRXO design

We have illustrated how the cluster randomisation aspect of the CRXO design leads to the formation of clusters of patients defined by ICU, while the crossover aspect of the design leads further to the formation of *cluster-periods* of patients within each cluster.

We have also illustrated how the cluster randomisation and crossover aspects of the CRXO design can lead to three sources (or components) of variation in the responses of patients in a CRXO trial: variation in the mean LOS between ICUs; variation in the mean LOS between cluster-periods; and variation between individual patient LOS within a cluster-period.

### The within-cluster within-period correlation and the within-cluster between-period correlation

In this section we show how the three sources of variation outlined in the preceding section can be used to quantify the similarity in LOS between the groups of patients defined by ICU (cluster) and cluster-period.

The *within-cluster within-period correlation (WPC)* quantifies the similarity of outcomes from patients in the same cluster-period. The *within-cluster between-period correlation (BPC)* quantifies the similarity of outcomes from patients in the same cluster, but in different periods. Specification of these two correlations are required to perform sample size estimates for a CRXO trial.

In the hypothetical circumstance where the LOS of an infinite number of patients admitted to each ICU is measured, we can determine the true WPC and BPC. In practice, the LOS can only be measured on a sample of patients, and the true WPC and BPC will be estimated from this sample of patients, with some amount of random sampling error.

We first describe the sources of variation underlying the BPC, and then extend the description to the WPC.

### Within-cluster between-period correlation (BPC)

The BPC measures how much of the total variability in the LOS is due to variability in the ICU mean LOS or analogously how similar patient responses are within the same cluster, but in different periods. The formula for the BPC, *η*, is:

1 $$ \eta =\frac{\sigma_C^2}{\sigma_C^2+{\sigma}_{CP}^2+{\sigma}_I^2}, $$

where *σ*_*C*_^2^ is the variance in mean LOS between clusters (ICUs), *σ*_*CP*_^2^ is the variance in mean LOS between cluster-periods, and *σ*_*I*_^2^ is the variance in individual LOS within a cluster-period.

The BPC measures the similarity between the LOS of two patients from the same ICU with one patient from the first period (cluster-period C) and one patient from the second period (cluster-period D).

The *similarity* between the LOS of patients in an ICU *between* cluster-periods arises from the *variability* in the ICU mean LOS *only*. We now refer to Fig. 2 to describe how this relationship between similarity and variability arises. As the ICU mean LOS (*red lines/red circles*) become more dispersed between ICUs, relative to the dispersion (i.e. distance) between cluster-period mean LOS within an ICU (*green lines/green circles*), the distribution of the patient LOS (*yellow/blue curves*) in the cluster-periods A and B become more similar to each other, as do the distribution of patient LOS in cluster-periods C and D.

For example, in Fig. 2c there is little variation in the ICU mean LOS around the overall mean LOS (*black line*) and the distribution of patient LOS in cluster-periods A, B, C and D almost all coincide. As a result, the similarity between the LOS of patients in different cluster-periods *within the same ICU* (e.g. one patient from cluster-period A and one patient from cluster-period B) is comparable to the similarity between the LOS of patients in different ICUs (e.g. one patient from cluster-period A and one patient from cluster-periods C or D). In contrast, in Fig. 2d, there is more separation between the ICU mean LOS and only the distributions of patient LOS from the same ICUs coincide (i.e. cluster-periods A and B, and cluster-periods C and D, coincide). As a result, the LOS of patients in different cluster-periods *within the same ICU* (e.g. one patient from cluster-period A and one patient from cluster-period B) are more similar to each other than to the patients in other ICUs (e.g. one patient from cluster-period A and one patient from cluster-periods C or D). Hence, the BPC is larger in Fig. 2d than in Fig. 2c. The same comparison can be made between Fig. 2g and h.

### The within-cluster within-period correlation (WPC)

The WPC measures how much of the total variability in the LOS is due to variability in the ICU mean LOS and the cluster-period mean LOS or analogously how similar patient responses are within a cluster-period. The formula for the WPC, *ρ*, is:

2 $$ \rho =\frac{\sigma_C^2+{\sigma}_{CP}^2}{\sigma_C^2+{\sigma}_{CP}^2+{\sigma}_I^2}. $$

The WPC measures the similarity in the LOS from two patients in the same cluster-period, e.g. cluster-period C.

The *similarity* between the LOS of patients *within* a cluster-period arises from the *variability* in the ICU mean LOS *and* cluster-period mean LOS. We now refer to Fig. 2 to describe how this relationship between similarity and variability arises. We describe the relationship in two parts: variability in the ICU mean LOS; and variability in the cluster-period mean LOS.

As the ICU mean LOS (*red circles/red lines*) becomes more disperse, relative to the dispersion (i.e. distance) between the cluster-period mean LOS (*green circles/green lines*), the distribution of the individual patient LOS (*yellow/blue curves*) in the four cluster-periods A, B, C and D become more distinct from each other, and hence patients within a cluster-period appear more similar to each other. For example, in Fig. 2c there is little variation between the ICU mean LOS around the overall mean LOS (*black line*) and the distribution of patient LOS in cluster-periods A, B, C and D almost all coincide. As a result, the similarity between the LOS of two patients in cluster-period A is comparable to the similarity between the LOS of one patient from cluster-period A and one patient from cluster-period B (or C or D). In contrast, in Fig. 2d, there is more separation between the ICU mean LOS and hence more separation of the patient LOS in ICUs 1 and 2. As a result, the LOS of two patients in cluster-period A are more similar to each other than to one patient from cluster-period A (cluster 1) and another patient from cluster-periods C or D (cluster 2). Hence, the WPC is smaller in Fig. 2c than in Fig. 2d. We note that the same comparison can be made between Fig. 2g and h.

Likewise, as the cluster-period mean LOS (*green circles/green lines*) becomes more disperses, relative to the distance between the ICU mean LOS (*red circles/red lines*), the distribution of the individual patient LOS (*yellow/blue curves*) in the four cluster-periods A, B, C and D also become more distinct from each other, and hence patients within a cluster-period become more similar to each other. For example, in Fig. 2d there is little variation between the cluster-period mean LOS around the ICU mean LOS and thus the distribution of patient LOS in cluster-periods A and B (and equivalently C and D) almost coincide. As a result, the similarity between the LOS of two patients in cluster-period A is comparable to the similarity between the LOS of one patient from cluster-period A and one patient from cluster-period B. In contrast, in Fig. 2h, there is more separation between the cluster-period mean LOS and the distribution of patient LOS. As a result, the LOS of two patients in cluster-period A are more similar to each other than to one patient from cluster-period A and another patient from cluster-period B (and even more similar than one patient from cluster-period A and another patient from cluster-periods C or D). Hence the WPC is again smaller in Fig. 2d than in Fig. 2h. We note that the same comparison can be made between Fig. 2c and g.

### Precision of the CRXO design compared to the parallel-group cluster randomised design and parallel-group, individually randomised design

In this section, we discuss how the WPC and BPC affect the precision of the estimate of the difference between interventions, and hence the sample size requirement, in a two-period, two-intervention, cross-sectional CRXO trial. We illustrate the two extremes of the CRXO design: when the precision in the CRXO design is equivalent to an IRCT design; and equivalent to a CRCT design. The allocation of interventions to patients in the IRCT, CRCT, and CRXO design are shown in Fig. 1.

**Fig. 3:**
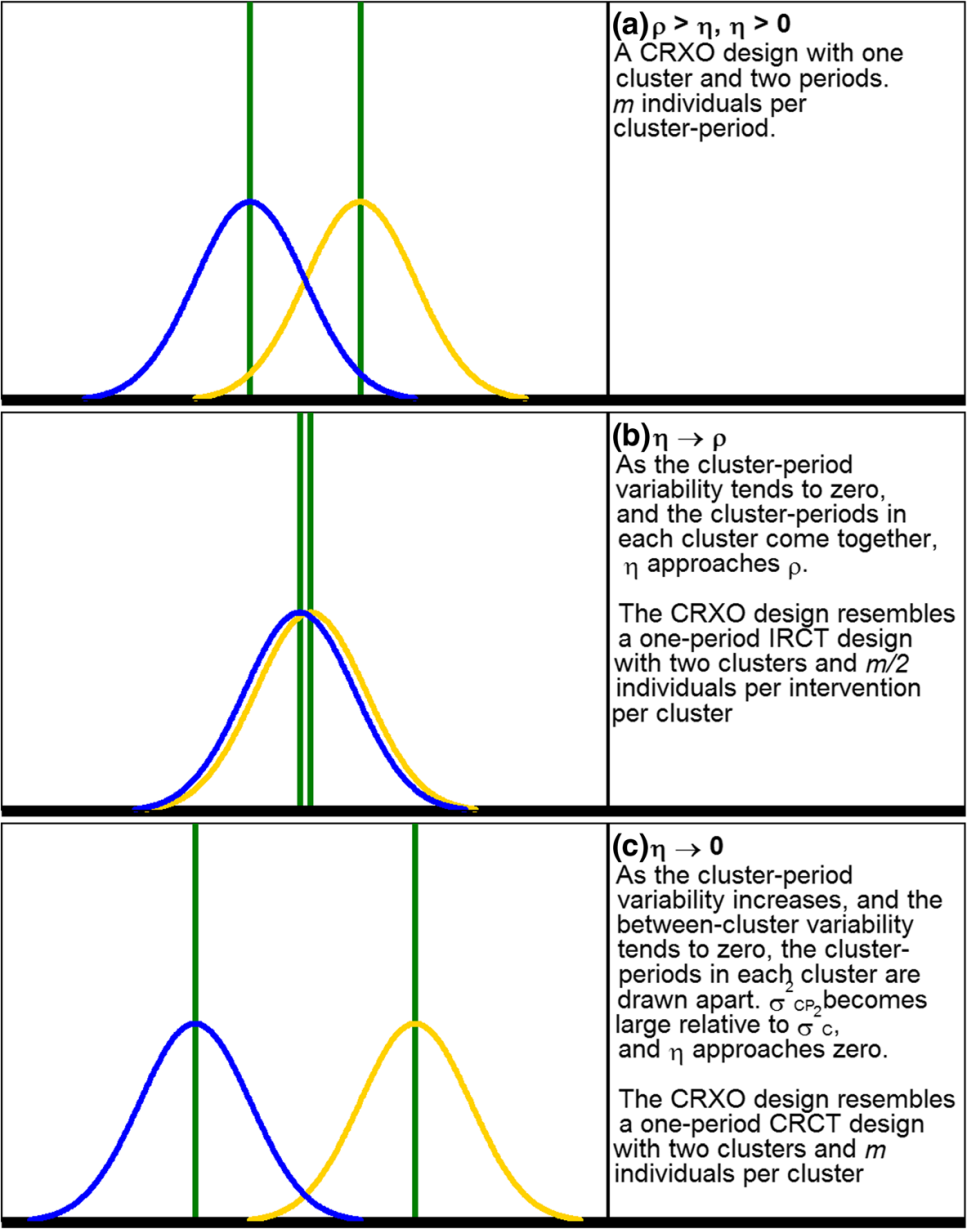
A single cluster in the cluster randomised crossover (CRXO) design where (**a**) *ρ* > *η*, *η* > 0. **b** *η* → *ρ*. **c** *η* → 0. The *green solid vertical lines* indicate difference between true intensive care unit (ICU) mean length of stay (LOS) and true cluster-period mean LOS. The *yellow (blue) curve* indicates a normal distribution of patient LOS within each cluster or cluster-period where the patient or cluster was allocated to intervention S (T). The true difference between intervention S and T is zero. The total variance in LOS remains constant

To illustrate the effect of the WPC and BPC on precision (and equivalently the components of variation), we continue to assume that the true difference between interventions is zero. We consider a large sample of patients admitted to *one* cluster in a CRXO design, such that the sampling error in the estimated mean LOS for patients is assumed negligible. Therefore, in the single cluster shown in Fig. 3, the separation between the distribution of LOS from patients receiving intervention S (*yellow curve*) and intervention T (*blue curve*) arises solely from the variation in the mean LOS between cluster-periods (*σ*_*CP*_^2^). In this section, we show which partitioning of the total variation in LOS into the components of variation leads to the most precision and to the least precision in the CRXO design.

In the CRXO design, the observed mean LOS of patients receiving each intervention can be compared within each cluster because each intervention is delivered in each cluster. As an illustration, in Fig. 3a, the observed difference in mean LOS between patients receiving each intervention could be due to a difference in true cluster-period mean LOS (*green lines*) but not due to differences in the true ICU mean LOS because this component of variation is removed when the two interventions are compared within an ICU.

As the variation in the true cluster-period mean LOS increases, and hence the separation between the *green lines* in Fig. 3a increases, the separation between the *yellow and blue curves* within an ICU increases. Correspondingly, from Eqs. 1 and 2, the difference between the WPC and BPC increases. In conclusion, increasing variability in the cluster-period means leads to increasing uncertainty in the observed difference in the mean LOS between patients receiving each intervention.

In the CRXO design, precision is maximised when there is no variation in LOS between periods within a cluster. In this scenario the separation between the *green lines* in Fig. 3a shrinks and the *yellow and blue curves* coincide, yielding Fig. 3b. The LOS of two patients in the same cluster-period are as similar as the LOS of two patients from the same ICU but in different cluster-periods. Also, from Eqs. 1 and 2, the WPC equals the BPC. Figure 3b now approximates the diagram that one would expect from an IRCT with two ICUs (with the mean LOS for each centre indicated by the *green lines*) and half the patients within each cluster receiving each intervention. This diagram arises in an IRCT because, for large sample sizes and under the assumption of no true differences between interventions, randomisation ensures that the distributions of LOS in each intervention (*yellow and blue curves*) are identical. The CRXO design will, therefore, have the same precision as an IRCT design.

Conversely, the precision of the CRXO design decreases when the cluster-period variability increases. As the variability between periods within a cluster increases, the separation between the *green lines*, and correspondingly the *yellow and blue curves*, in Fig. 3a increases. The increased separation results in greater variability in the comparison of patient LOS in each intervention within each cluster. For a fixed total variability in ICU LOS, as the variability between periods within a cluster increases, the variability between *different* clusters must reduce. In the limiting case there is no variation at all between clusters (*σ*_*C*_^2^ = 0), resulting in the BPC equalling zero (Eq. 1). In this case each cluster-period effectively resembles a separate cluster (Fig. 3c). Two patients in different cluster-periods *in the same ICU* are no more similar than two patients *in different ICUs*. Therefore, there is no advantage to the crossover component of the CRXO design and the CRXO will have the same precision as a CRCT design.

In most situations, the BPC will lie between zero and the WPC. In the following section, ‘Performing a sample size calculation’, we discuss the effect of the BPC and WPC on the sample size required to be able to detect a specified true intervention effect in a CRXO trial with a given level of power, and provide guidance on how to choose values for the BPC and WPC for a sample size calculation.

## Performing a sample size calculation

The sample size required to detect a specified true difference between interventions with a given level of power decreases as the precision of the estimate of the intervention effect increases. In the ‘Understanding the CRXO design’ section, we considered precision in the CRXO design when the true difference between interventions was assumed to be zero. However, even when the true difference is not zero, the effects of the WPC and BPC on precision described in the previous section continue to hold.

The sample size required for a CRXO trial increases as the cluster-period variability increases, or equivalently as the difference between the WPC and BPC increases. As the value of the BPC increases from zero to the WPC, the sample size required for the CRXO design will decrease from that required for a CRCT design towards the sample size for an IRCT. Therefore, using an appropriate specification of the difference between the WPC and the BPC is essential for performing sample size calculations for the CRXO design.

We now illustrate how to perform a sample size calculation for a two-period, two-intervention CRXO trial with a continuous and binary outcome using ICU LOS and in-ICU mortality data, respectively, from the Australian and New Zealand Intensive Care Society (ANZICS) Adult Patient Database (APD) [14, 15]. There are 37 tertiary ICUs in Australia and New Zealand, of which 25 to 30 might be expected to participate in a trial.

We compare the sample size requirement for number of individuals and number of clusters (ICUs) from the CRXO design with the requirement from the stratified, multicentre, parallel-group, individually randomised design (IRCT) and the parallel-group cluster randomised design (CRCT) conducted over one period.

Comparisons of the sample size requirements for these different designs can either be made by fixing the total number of clusters across all designs; or by treating the CRXO design as lasting twice as long, i.e. two periods, instead of one period as in the IRCT and CRCT designs. We take the latter approach here so that the WPC is the same in each period.

We include Stata do-files to estimate the required sample size for each trial design, for a chosen set of sample size parameters (see Additional files 1 and 2).

### The sample size formulae for a one-period IRCT design, a one-period CRCT design, and a two-period, two-intervention, cross-sectional CRXO design

The sample size formula for the *total number of participants* required for a normally distributed continuous outcome in a two-period, two-intervention CRXO trial, across all clusters and interventions, assuming a constant number of participants recruited to each cluster-period is [8]:

$$ {N}_{CRXO}=2\ {\left({z}_{\alpha /2}+{z}_{\beta}\right)}^2\frac{2{\sigma}^2}{{\left({\mu}_A-{\mu}_B\right)}^2}\ \left(1+\left(m-1\right)\rho -m\ \eta \right)+4m, $$

and for a one-period, two-intervention CRCT:

$$ {N}_{CRCT}=2\ {\left({z}_{\alpha /2}+{z}_{\beta}\right)}^2\frac{2{\sigma}^2}{{\left({\mu}_A-{\mu}_B\right)}^2\ }\ \left(1+\left(m-1\right)\rho \right)+2m, $$

and for a one-period, two-intervention, parallel-group IRCT, stratified by cluster, across all clusters and interventions is [16]:

$$ {N}_{IRCT}=2\ {\left({z}_{\alpha /2}+{z}_{\beta}\right)}^2\frac{2{\sigma}^2}{{\left({\mu}_A-{\mu}_B\right)}^2\ }\ \left(1-\rho \right), $$

where z_α/2_ and z_β_ are the standard normal values corresponding to the upper tail probabilities of *α*/2 and *β*, respectively; *α* is the two-sided significance level, typically 0.05; 1 − *β* is the power to detect the specified difference (μ_A_ − μ_B_) with probability α; σ^2^ is the variance of the outcome; *μ*_*A*_ and *μ*_*B*_ are the outcome means in each arm; *m* is the number of participants per cluster-period; *ρ* is the WPC; and *η* is the BPC.

The formulae presented above include a correction for when the number of clusters small, as suggested in Eldridge and Kerry (p. 149) [2] and Forbes et al. [9]. This leads to an additional 4 *m* participants in the CRXO design and 2 *m* participants in the CRCT design. No correction is necessary for the IRCT because the number of individual participants will be large in the example settings.

For a binary outcome we can replace $\frac{2{\sigma}^2}{{\left({\mu}_A-{\mu}_B\right)}^2}$ with $\frac{p_A\left(1-{p}_A\right)+{p}_B\left(1-{p}_B\right)}{{\left({p}_A-{p}_B\right)}^2\kern1.25em }$ in the above formulae [12], where *p*_*A*_ and *p*_*B*_ are the proportions of the outcomes in each arm.

For the CRXO design, CRCT design and IRCT design, respectively, the formulae to determine the number of clusters (ICUs) needed to achieve the required number of participants are:

$$ {n}_{CRXO}=\frac{N_{CRXO}}{2m} $$, $$ {n}_{CRCT}=\frac{N_{CRCT}}{m} $$, and $$ {n}_{IRCT}=\frac{N_{IRCT}}{m} $$_._

### Australian and New Zealand Intensive Care Society – Adult Patient Database (ANZICS-APD): estimates of the WPC and BPC

The ANZICS-APD is one of four clinical quality registries run by the ANZICS Centre for Outcome and Resource Evaluation and collects de-identified information on admissions to adult ICUs in Australia and New Zealand. A range of data is collected during patients’ admissions, including ICU LOS and in-ICU mortality. In this section we use the ANZICS-APD data from 34 tertiary ICUs to estimate the correlations required to perform sample size calculations for CRXO trials. We estimate the values of the WPC and the BPC from two 12-month periods of data between 2012 and 2013 (Appendix 1).

#### Continuous outcomes

We follow the methods of Turner et al. to estimate the WPC and BPC (Appendix 1). Using the ICU LOS data, the estimated WPC was $\widehat{\rho}\kern0.5em =\kern0.5em 0.038$, and the BPC was $\widehat{\eta}\kern0.5em =\kern0.5em 0.032$ (Table 1). The overall mean LOS was 5.3 log-hours, with a standard deviation 1.39 log-hours.

**Table 1:** Calculation of the within-cluster, within-period correlation (WPC) and within-cluster, between-period correlation (BPC) for intensive care unit (ICU) log-length of stay (LOS) in the Australian and New Zealand Intensive Care Society – Adult Patient Database (ANZICS-APD)

$$ {\widehat{\sigma}}_{ICU}^2=0.045 $$	
$$ {\widehat{\sigma}}_{CP}^2=0.008 $$	
$$ {\widehat{\sigma}}_I^2=1.360 $$	
$$ \widehat{\rho}=\frac{{\widehat{\sigma}}_{ICU}^2+{\widehat{\sigma}}_{CP}^2}{{\widehat{\sigma}}_{ICU}^2+{\widehat{\sigma}}_{CP}^2+{\widehat{\sigma}}_I^2}\kern0.5em =\kern0.5em \frac{0.045+0.008}{0.045+0.008+1.360}\kern0.5em =\kern0.5em 0.038 $$	
$$ \widehat{\eta}=\frac{{\widehat{\sigma}}_{ICU}^2}{{\widehat{\sigma}}_{ICU}^2+{\widehat{\sigma}}_{CP}^2+{\widehat{\sigma}}_I^2}\kern0.5em =\kern0.5em \frac{0.045}{0.045+0.008+1.360}\kern0.5em =\kern0.5em 0.032 $$	

#### Binary outcomes

We follow the methods of Donner et al. to estimate the WPC and BPC (Appendix 1). Using the in-ICU mortality data, the estimated WPC was $\widehat{\rho}=0.010$, and the BPC was $\widehat{\eta}=0.007$. The overall mortality rate was 8.7%.

### Sample size example for ICU LOS

Suppose we wish to design a two-period, two-intervention, CRXO trial to have 80% power to detect a true reduction in ICU LOS of 0.1 log-hours (1.1 h) using a two-sided test with a Type-I error rate of 5%. In practice, the choice of reduction in ICU LOS should be the minimally clinically important reduction, determined in consultation with subject matter experts. A 0.1 log-hours’ reduction is equivalent to a 10% reduction, and is a reasonable minimally clinically important reduction in ICU LOS.

The standard deviation is estimated to be 1.2 log-hours (3.3 h). As an illustration, we assume that in a 12-month period, 200 patients in each ICU will meet the inclusion criteria for the trial. The CRXO trial will, therefore, run for 2 years and include 400 patients per ICU, with 200 patients receiving each intervention in each ICU.

For comparison, we consider an IRCT and a CRCT run for a 12-month period, with 100 patients receiving each intervention in each ICU in the IRCT and all 200 patients receiving one intervention in each ICU in the CRCT.

Using the estimates that we calculated from the ANZICS-APD data for the WPC and BPC, the total number of patients and ICUs for each design are summarised in Table 2 (see Appendix 2 for calculations).

**Table 2:** Number of individuals and number of clusters required for a cluster randomised crossover (CRXO), cluster randomised controlled trial (CRCT) and individually randomised controlled trial (IRCT) trial with *ρ* = 0.038 for all designs and specified *η* for CRXO design

	Number of required individuals	Number of required ICUs
CRXO		
*ρ* = 0.038, *η* = 0.032	10,564	27
*ρ* = 0.038, *η* = 0.010	30,433	77
CRCT	39,065	196
IRCT	4345	22

*ICU* intensive care unit

**Table 3:** Number of individuals and number of clusters required for a cluster randomised crossover (CRXO), cluster randomised controlled trial (CRCT) and individually randomised controlled trial (IRCT) trial with *ρ* = 0.010 for each design and specified *η* for the CRXO design

	Number of required individuals	Number of required ICUs
CRXO		
*ρ* = 0.010, *η* = 0.007 (equal cluster sizes)	51,581	22
*ρ* = 0.010, *η* = 0.006 (equal cluster sizes)	63,811	27
*ρ* = 0.010, *η* = 0.007 (unequal cluster sizes)	41,208	23
CRCT	13,4792	113
IRCT	10,090	9

*ICU* intensive care unit

The total number of participants required for the CRXO design is *N*_*CRXO*_ = 10,564. To include 10,564 participants, we require *n*_*CRXO*_ = 27 ICUs, each recruiting 200 participants in each of the *two* 12-month periods. If instead we conducted a CRCT over a *single* 12-month time period, the total number of participants required would be *N*_*CRCT*_ = 39,065. Assuming that 200 patients are eligible in each ICU, we would need *n*_*CRCT*_ = 196 ICUs. The total number of participants required for an IRCT conducted over a 12-month period is *N*_*IRCT*_ = 4345. With 200 patients per ICU (100 patients per intervention), the total number of ICUs required is *n*_*IRCT*_ = 22.

In this example, the CRXO design required five more clusters (ICUs) than the IRCT design; however, the CRXO design is run for twice as long. The CRCT design would require 7.3 times as many clusters as the CRXO design. Given that there are only 37 tertiary ICUs in Australia and New Zealand, a CRCT trial would not be feasible.

We can examine the sensitivity of the CRXO sample size calculation to a different BPC. If the BPC was *η* = 0.010 rather than *η* = 0.032, then the CRXO design requires *N*_*CRXO*_ = 30,433 participants. The total number of ICUs required to obtain the required number of participants is *n*_*CRXO*_ = 77. The total number of ICUs required has now increased by 50, and the trial would no longer be feasible in the Australia and New Zealand region within tertiary ICUs only. Note that when the number of patients admitted in each cluster-period is relatively large, we would observe a similar increase in the sample size if we had underestimated the WPC by 0.023, rather than overestimated the BPC by 0.023.

### Sample size example for in-ICU mortality

In a second example, suppose that we wish to design a study to have 80% power to detect a true reduction in in-ICU morality from 8.7% to 7.2% (absolute difference of 1.5%) using a two-sided test with a Type-I error rate of 5%. From the ANZICS-APD admission data, we estimate that in a 12-month period, 1200 patients will be admitted in each ICU and eligible for inclusion in the trial. The total number of patients and ICUs for each design are summarised in Table 3 (see Appendix 2 for calculations).

For a CRXO design, using the estimates for the WPC, the BPC, and the cluster-period size we calculated from the ANZICS-APD, the total number of participants required is *N*_*CRXO*_ = 51,581. Since we expect 1200 patients in each ICU for each of the two 12-month periods, the required number of ICUs is *n*_*CRXO*_ = 22. If we had used a CRCT, the required number of participants is *N*_*CRCT*_ = 134, 792. Assuming that 1200 patients admitted over a single 12-month period, we would need *n*_*CRCT*_ = 113 ICUs. The total number of participants required for the IRCT design is *N*_*IRCT*_ = 10,090. For a trial run over 12 months, with 1200 patients per ICU (600 patients per intervention), the total number of ICUs required is *n*_*IRCT*_ = 9.

In this example, the CRXO design required 2.4 times as many clusters (ICUs) as the IRCT design, and is run for twice as long. Despite the increase in required clusters, the CRXO is still a feasible design, unlike the CRCT design, which would require 5.1 times as many clusters as the CRXO design.

We can examine the sensitivity of the CRXO sample size calculation to a different BPC. If the BPC was *η* = 0.006, rather than *η* = 0.007, then the total number of participants required is *N*_*CRXO*_ = 63,811. Since we expect 1200 patients for each cluster-period, we would need to include *n*_*CRXO*_ = 27 ICUs, i.e. 54 cluster-periods. This demonstrates that a small change in the assumed BPC can have a marked impact on the number of required ICUs and patients.

### Unequal cluster-period sizes

We have so far assumed that the cluster-period size is constant. In reality, it is likely that different ICUs will include a differing number of participants [17, 18]. An extension to the sample size formula for this scenario is provided by [9]. When the analysis is based on unweighted cluster-period means, the arithmetic mean in the sample size formula given for the CRXO design can be replaced by the harmonic mean:

$$ {m}_h=n{\sum}_{i=1}^n\frac{1}{m_i}. $$

We assume that the cluster-period size is the same in each period within a cluster. For further extensions, see Forbes et al. [9].

From the ANZICS-APD data, we estimate that the harmonic mean is *m*_*h*_ = 900. Therefore then the required number of patients is *N*_*CRXO*_ = 41,208, and the required number of ICUs is:

$$ {n}_{CRXO}=\frac{41208}{2\times 900}=23. $$

Allowing for unequal cluster-period sizes has increased the required number of clusters slightly from 22 to 23.

### Guidance on how to choose the WPC and the BPC for the sample size calculation

As was seen in the ‘Understanding the CRXO design’ section, the difference between the WPC and BPC is key in determining the sample size for a CRXO design.

Approaches for choosing the within-cluster intracluster correlation (ICC) in sample size calculations for parallel-group CRCTs have been discussed [19–22]. Similar considerations apply when choosing the WPC in a CRXO design. In particular, because the ICC estimates are subject to large uncertainty [23], reviewing multiple relevant estimates of the ICC is recommended. These ICC estimates may be obtained from trial reports, lists published in journal articles or from routinely collected data.

Identification of the factors which influence the magnitude of the within-cluster ICC can assist trialists in selecting ICC estimates that are relevant to their planned trial. Typically, the trial outcome itself is less predictive of the value of the ICC than factors such as: the type of outcome variable (i.e. process outcomes that measure adherence to protocol and policy or individually measured outcomes) [19], the prevalence of the outcome [20], the size of the natural cluster of individuals that the randomised clusters are formed from [20], and the characteristics of the individuals and clusters [22].

The duration of time over which the outcome variables were measured may also affect the value of the within-cluster ICC. As the measurements of individuals within a cluster become further apart, the similarity between the measurements might be expected to decrease. Using an estimate of the within-cluster ICC that was determined over a different duration of time than the intended period length of the planned trial assumes that there is no variation in the within-cluster ICC over time, and we are unaware of any research investigating if this is justified.

In contrast, we are aware of only two publications reporting estimates of the BPC [24, 25]. Therefore, until reporting of the BPC becomes more common [26], estimates of the BPC are likely to rely on the analysis of routinely collected data, pilot or feasibility study data, or a reasoned best-guess. As for the within-cluster ICC in cluster randomised trials, estimating the BPC from feasibility or a single routinely collected data source is likely to be subject to considerable uncertainty [27].

In forming a best guess, it is helpful to recognise that the difference between the WPC and BPC is a measure of changes over time within a cluster’s environment that affect the outcomes of each individual in that cluster (e.g. a change in policy in one ICU). Over short time periods or in clusters with stable environments and patient characteristics, it might be reasonable to expect little change over time and, therefore, the BPC will be similar to the WPC. However, if this assumption is untrue and the BPC is less that the WPC, a sample size calculation assuming that the two correlations are equal will lead to an underpowered study. It may be prudent to assume that the BPC is less than the WPC. To this end, suggestions have been made to set the BPC to: half the WPC [12]; and to 0.8 of the WPC [11].

In the ANZICS-APD the ratio of the BPC to WPC is 0.7 for ICU mortality and 0.8 for ICU LOS, which is consistent with the suggestion made by Hooper and Bourke [11]. In the absence of multiple estimates or precise estimates of the ICCs, a conservative approach in selecting the BPC is recommended to avoid an underpowered trial. Further, a sensitivity analysis exploring the effect of the choice of ICC on the sample size is recommended.

## Common mistakes when performing sample size calculations and analyses

Many trialists have made strong assumptions about the values of the WPC and the BPC in their sample size and analysis methodology [13]. In this section we illustrate the consequences of using incorrect sample size methodology on the estimated sample size and power.

### Assume the outcomes are independent

In a review of CRXO trials, 34% of sample size calculations made the assumption that the observations were independent [13]. There are two scenarios where this assumption is reasonably appropriate: when the WPC and the BPC are equal and the sample size calculation was stratified by centre; or when the WPC and the BPC are both zero.

The first scenario arises when the outcomes of two individuals in the same cluster are equally similar if the individuals are in different periods as if the individuals are in the same period (i.e. there is no change in the WPC over time within a cluster). In this fortuitous case the precision gained by crossover aspect of the CRXO design equals the precision lost by cluster randomisation (apart from a factor of 1-WPC, which is usually small [16]). The second scenario arises when there is no similarity between the outcomes of any two individuals, which is unlikely.

The effect on power of assuming that the outcomes are independent will depend on the cluster-period size and the difference between the WPC and the BPC. Loss of power will increase as both the difference between the two ICCs increases and the cluster-period size increases.

We illustrate the potential effect on power and sample size assuming the outcomes are independent using a published sample size calculation. Roisin [28] estimated that the seven wards (clusters) participating in their trial required a minimum of 3328 patients to have 80% power to detect a reduction in proportion of hospital acquisition of methicillin-resistant *Staphylococcus aureus* (MRSA) from 3% to 1.5%. From the ANZICS-APD data, we estimate a WPC of 0.010, and a BPC of 0.007 for in-ICU mortality in the ICU setting. As an example only, we assume that the estimates of the correlations for ICU mortality are similar to the correlations for ICU MRSA acquisition. Given that a total of 2505 patients were eligible for inclusion in the study, we determined the average cluster-period size to be 179. From these estimates, we determine that a sample size of 5385 is required to achieve the specified power, which is a 62% increase from the published sample size requirement of 3328.

### Assume a parallel-group cluster randomised design instead of a cluster randomised crossover design

Another common approach when performing sample size calculations for CRXO trials is to use methods designed for parallel-group CRCT trials. Applying CRCT sample size methodology to a CRXO design makes the assumption that: the BPC is zero; and that the WPC calculated over all periods in the trial is the same as the WPC calculated for a single period. Under the assumption that the BPC is zero, the outcomes of individuals within a cluster, but in different periods, are no more similar than outcomes of individuals in different clusters. That is, the individuals in different periods are assumed to be independent. When the BPC is not zero, the CRCT design effect does not account for the gain in precision achieved by the crossover aspect of the CRXO design, leading to a potentially overpowered trial. Trials that use CRCT sample size methods become progressively more overpowered as the true BPC becomes larger and the cluster-period sizes increase.

We illustrate the potential effect on power and the sample size requirement using CRCT sample size methodology by means of a published sample size calculation. van Duijn [29] estimated that eight ICUs (clusters) participating in their trial would include 135 patient measurements per cluster-period. Using CRCT sample size methodology, each of the 16 cluster-periods (two periods per ICU) were assumed to be separate clusters of 135 patients. van Duijn [29] assumed a within-cluster ICC of 0.01, and hence they estimated that the trial required 1842 patients to have 80% power to detect a reduction in proportion of ICU patients with antibiotic-resistant gram-negative bacteria from 55% to 45%. From the ANZICS-APD data, we estimate a WPC of 0.010, and a BPC of 0.007, as in the example in the previous section. From these estimates, we determine that a sample size of 1623 is required to achieve the specified power, which is 12% less than the sample size required for a CRCT.

## Discussion

Sample size calculations for CRXO trials need to account for both the cluster randomisation and crossover aspects of the design to ensure that an appropriate number of participants are recruited to adequately address the trial’s hypotheses. There are simple, sample size formulae available for a two-period, two-intervention, cross-sectional CRXO design; however, the implementation of these formulae has been limited [13]. Such limited use of the formula may be due to a lack of recognition that formulae are available, a lack of availability of estimates of the parameters required within the formulae, or a lack of trialists’ understanding of those parameters.

We have illustrated how the cluster randomisation and crossover aspects of the CRXO design give rise to similarity in both the responses of individuals within the same cluster and within the same cluster-period; and have described the parameters required to perform sample size calculations for CRXO trials. We have provided guidance on how to choose the parameters required for the sample size calculation and perform sample size calculation using those parameters.

While our focus has been on the two-intervention, two-period, cross-sectional CRXO design, more complex designs with additional periods and interventions are possible. The sample size and analysis methodology is more complex in these designs. For example, in a design with more than two periods, additional assumptions are required about the similarity between individuals in the same cluster in the same time period, and 1, 2, or 3, etc. time periods apart. Careful consideration should always be given to whether cluster randomisation is necessary [30], and whether the risk of the intervention effect from one period carrying over to the next period is minimal [6].

In addition to consideration of the sample size methodology, it is also essential to appropriately account for the cluster and the cluster-period in the analysis. Very few published trials do so [13]. Failure to account for the cluster-period in an individual level analysis leads to inflated Type-I error rates [31]. Methods to analyse CRXO trials have been published by Turner et al. and Forbes et al. [5, 9].

## Conclusions

Sample size calculations for CRXO trials must account for both the cluster randomisation and crossover aspects of the design. In this tutorial we described how the CRXO design can be understood in terms of components of variation in the individual outcomes, or equivalently, in terms of correlations between the outcomes of individual patients. We illustrated how to perform sample size calculations for continuous and binary outcomes, and provided guidance on selecting estimates of the parameters required for the sample size calculation.

### Additional files

Additional file 1: Continuous outcomes sample size Stata do file. Stata do file to perform sample size calculations for continuous outcomes using formulae presented in the ‘Performing a sample size calculation’ section, for a given set of sample size parameters. (DO 1 kb)

Additional file 2: Binary outcomes sample size Stata do file. Stata do file to perform sample size calculations for binary outcomes using formulae presented in the ‘Performing a sample size calculation’ section, for a given set of sample size parameters. (DO 2 kb)

## Appendix 1

### Estimates of the WPC and BPC

To illustrate the impact of the WPC and BPC on the sample size calculation, we estimate the values of the WPC and BPC by using previously published methods for continuous and binary outcomes [5, 12].

#### Continuous outcomes

ICU LOS is right-skewed, so we begin by log-transforming this variable, so that the assumptions of the model used to estimate the correlations are more likely to be met. We use LOS to represent log(LOS) throughout. We estimate the values of the WPC and the BPC from the variances estimated by fitting the following model [5]:

$$ {Y}_{ij k}=\mu +\pi +{u}_i+{v}_{ij}+{e}_{ij k}, $$

where there are *i* = 1, …, *n* ICUs, *j* = 1, 2 12-month periods and *k* = 1, …, *m*_*ij*_ patients in the *i* ^*th*^ ICU (cluster) and *j* ^*th*^ period; *Y*_*ijk*_ is the LOS for the *k* ^*th*^ patient in the *j* ^*th*^ cluster-period in the *i* ^*th*^ ICU (cluster); *μ* is the overall mean LOS; *π* is the fixed period effect; *u*_*i*_ ~ *N*(0, *σ*_*C*_^2^) is the difference from the overall mean LOS for each ICU mean LOS; *v*_*ij*_ ~ *N*(0, *σ*_*CP*_^2^) is the difference from the ICU mean LOS for each cluster-period mean LOS, and *e*_*ijk*_ ~ *N*(0, *σ*_*I*_^2^) is the difference from the cluster-period mean LOS for each patient LOS; *σ*_*C*_^2^, *σ*_*CP*_^2^, and *σ*_*I*_^2^ are the variances for the ICU (cluster) mean LOS, cluster-period mean LOS and patient LOS within each cluster-period, respectively.

Because we are fitting the model to registry data, rather than clinical trial data of the actual treatments to be considered, we estimate the model parameters under the assumption of a null treatment effect, and hence have not included a fixed treatment effect. A fixed treatment effect should be included when estimating the variance components from data from the actual clinical trial.

The model was fitted in Stata 14 with the mixed command using restricted maximum likelihood estimation: mixed log(LOS) periodeffect || cluster: || cluster_period:, reml.

#### Binary outcomes

We estimate the value of the WPC for within-ICU mortality by fitting the analysis of variance (ANOVA) estimator for the intracluster correlation [12]:

$$ \widehat{\rho}=\kern0.5em \frac{MSC- MSW}{MSC+\left({m}_0-1\right) MSW}, $$

$$ MSC=\frac{\sum_{j=1}^2{\sum}_{i=1}^n{m}_{ij}{\left({\widehat{P}}_{ij}-{\widehat{P}}_j\right)}^2}{\sum_{j=1}^2\left(n-1\right)}, $$

$$ MSW=\frac{\sum_{j=1}^2{\sum}_{i=1}^n{m}_{ij}{\widehat{P}}_{ij}\left(1-{\widehat{P}}_{ij}\right)}{\sum_{j=1}^2\left({N}_j-n\right)}, $$

$$ {m}_0=\frac{N-{\sum}_{j=1}^2{\sum}_{i=1}^n{m}_{ij}^2/{N}_j\ }{\sum_{j=1}^2\left(n-1\right)}, $$

where there are *i* = 1, …, *n* ICUs and *j* = 1, 2 12-month periods; *m*_*ij*_ is the number of patients in the *i* ^*th*^ ICU (cluster) and *j* ^*th*^ period; *N*_*j*_ is the total number of patients in each period and *N* is the total number of patients overall; ${\widehat{P}}_{ij}$ is the estimated mortality rate in each cluster-period; and ${\widehat{P}}_j$ is the estimated mortality rate in period *j*.

And by fitting the Pearson pairwise estimator for the BPC [12]:

$$ \widehat{\eta}=\frac{\sum_{i=1}^n\left({Y}_{1i}-{m}_{1i}{\widehat{P}}_1\right)\left({Y}_{2i}-{m}_{2i}{\widehat{P}}_2\right)}{\sqrt{\left({\sum}_{i=1}^n{m}_{2i}\left({Y}_{1i}-2{Y}_{1i}{\widehat{P}}_1+{m}_{1i}{\widehat{P}}_1^2\right)\left)\right({\sum}_{i=1}^n{m}_{1i}\left({Y}_{2i}-2{Y}_{2i}{\widehat{P}}_2+{m}_{2i}{\widehat{P}}_2^2\right)\right)}}, $$

where *Y*_1*i*_ and *Y*_2*i*_ are the number of deaths in two adjacent time periods on the *i* ^*th*^ ICU.

## Appendix 2

### Sample size calculations

In this section we provide the details of the sample size calculations presented in the ‘Performing a sample size calculation’ section, using the estimates for the WPC and BPC that we calculated from the ANZICS-APD data in Appendix 1.

#### Sample size calculation for ICU LOS

*Total number of participants and ICUs required for the CRXO design*

$$ {N}_{CRXO}=2\ {\left({z}_{\alpha /2}+{z}_{\beta}\right)}^2\frac{2{\sigma}^2}{{\left({\mu}_A-{\mu}_B\right)}^2}\ \left(1+\left(m-1\right)\rho -m\ \eta \right)\kern0.5em +\kern0.5em 4m, $$

$$ {N}_{CRXO}=2\times {\left(1.96+0.84\right)}^2\frac{2\times {1.2}^2}{{\left(5.3-5.2\right)}^2}\left(1+\left(200-1\right)0.038-200\times 0.032\right)+4\times 200=10564 $$

Since we expect 200 patients in each ICU for each of the two 12-month periods, the number of ICUs needed to achieve the required number of participants is:

$$ {n}_{CRXO}=\frac{N_{CRXO}}{2m}=\frac{10564}{2\times 200}=27. $$

If the BPC was *η* = 0.010 rather than *η* = 0.032, then:

$$ {N}_{CRXO}=2\times {\left(1.96+0.84\right)}^2\frac{2\times {1.2}^2}{{\left(5.3-5.2\right)}^2}\left(1+\left(200-1\right)0.038-200\times 0.010\right)+4\times 200=30433 $$

The total number of ICUs required to obtain the required number of participants is:

$$ {n}_{CRXO}=\frac{N_{CRXO}}{2m}=\frac{30433}{2\times 200}=77. $$

*Total number of participants and ICUs required for the CRCT design*

$$ {N}_{CRCT}=2\ {\left({z}_{\alpha /2}+{z}_{\beta}\right)}^2\frac{2{\sigma}^2}{{\left({\mu}_A-{\mu}_B\right)}^2\ }\ \left(1+\left(m-1\right)\rho \right)+2m, $$

$$ {N}_{CRCT}=2\ {\left(1.96+0.84\right)}^2\frac{2\times {1.2}^2}{{\left(5.3-5.2\right)}^2}\left(1+\left(200-1\right)0.038\right)+2\times 200=39065 $$

Assuming that 200 patients are eligible in each ICU over the 12-month trial period, we would need to include:

$$ {n}_{CRCT}=\frac{N_{CRCT}}{m}=\frac{39065}{200}=196\kern0.5em \mathrm{ICUs}. $$

*Total number of participants and ICUs required for the IRCT design*

$$ {N}_{IRCT}=2\ {\left({z}_{\alpha /2}+{z}_{\beta}\right)}^2\frac{2{\sigma}^2}{{\left({\mu}_A-{\mu}_B\right)}^2\ }\ \left(1-\rho \right), $$

$$ {N}_{IRCT}=2\ {\left(1.96+0.84\right)}^2\frac{2\times {1.2}^2}{{\left(5.3-5.2\right)}^2}\left(1-0.038\right)=4345. $$

For a trial run over 12 months, with 200 patients per ICU (100 patients per intervention), the total number of ICUs required is:

$$ {n}_{IRCT}=\frac{N_{IRCT}}{m}=\frac{4345}{200}=22. $$

#### Sample size calculation for in-ICU mortality

*Total number of participants and ICUs required for the CRXO design*

$$ {N}_{CRXO}=2\times {\left({z}_{\alpha /2}+{z}_{\beta}\right)}^2\frac{p_A\left(1-{p}_A\right)+{p}_B\left(1-{p}_B\right)}{{\left({p}_A-{p}_B\right)}^2\kern1.25em }\ \left(1+\left(m-1\right)\rho -m\ \eta \right)+4m, $$

$$ {\displaystyle \begin{array}{l}{N}_{CRXO}=2\times {\left(1.96+0.84\right)}^2\frac{0.087\times \left(1-0.087\right)+0.072\times \left(1-0.072\right)}{{\left(0.087-0.072\right)}^2\kern1.25em }\ \left(1+\left(1200-1\right)\right.\\ {}\kern8.5em \left.\times 0.010-1200\times 0.007\right)+4\times 1200\kern0.5em =\kern0.5em 51581\end{array}} $$

The number of ICUs needed to achieve the required number of participants is:

$$ {n}_{CRXO}=\frac{N_{CRXO}}{2m}=\frac{51581}{2\times 1200}=22. $$

If the BPC was *η* = 0.006, rather than *η* = 0.007, then the total number of participants required is:

$$ {\displaystyle \begin{array}{l}{N}_{CRXO}=2\times {\left(1.96+0.84\right)}^2\frac{0.087\times \left(1-0.087\right)+0.072\times \left(1-0.072\right)}{{\left(0.087-0.072\right)}^2\kern1.25em }\ \left(1+\left(1200-1\right)\times 0.010-1200\times 0.006\right)\\ {}\kern11.5em +4\times 1200=63811\end{array}} $$

We would need to include:

$$ {n}_{CRXO}=\frac{N_{CRXO}}{2m}=\frac{63811}{2\times 1200}=27\kern0.5em \mathrm{ICUs}. $$

*Total number of participants and ICUs required for the CRCT design*

$$ {N}_{CRCT}=2\ {\left({z}_{\alpha /2}+{z}_{\beta}\right)}^2\frac{p_A\left(1-{p}_A\right)+{p}_B\left(1-{p}_B\right)}{{\left({p}_A-{p}_B\right)}^2\kern1.25em }\ \left(1+\left(m-1\right)\rho \right)+2m, $$

$$ {N}_{CRCT}=2\ {\left(1.96+0.84\right)}^2\frac{0.087\times \left(1-0.087\right)+0.072\times \left(1-0.072\right)}{{\left(0.087-0.072\right)}^2\kern1.25em }\ \left(1+\left(1200-1\right)\times 0.010\right)+2\times 1200=134792 $$

We would need ${n}_{CRCT}=\frac{N_{CRCT}}{m}=\frac{134792}{1200}=113\kern0.5em \mathrm{ICUs}.$

*Total number of participants and ICUs required for the IRCT design*

$$ {N}_{IRCT}=2\ {\left({z}_{\alpha /2}+{z}_{\beta}\right)}^2\frac{p_A\left(1-{p}_A\right)+{p}_B\left(1-{p}_B\right)}{{\left({p}_A-{p}_B\right)}^2}\kern1.25em \left(1-\rho \right), $$

$$ {N}_{IRCT}=2\ {\left(1.96+0.84\right)}^2\frac{0.087\times \left(1-0.087\right)+0.072\times \left(1-0.072\right)}{{\left(0.087-0.072\right)}^2}\kern1.25em \left(1-0.010\right)=10090 $$

The total number of ICUs required is:

$$ {n}_{IRCT}=\frac{N_{IRCT}}{m}=\frac{10090}{1200}=9. $$

## Abbreviations

ANZICS-APD: Australia and New Zealand Intensive Care Society – Adult Patient Database
BPC: Within-cluster between-period correlation
CRCT: Cluster randomised controlled trial
CRXO: Cluster randomised crossover
ICC: Intracluster correlation
ICU: Intensive care unit
IRCT: Individually randomised controlled trial
LOS: Length of stay
WPC: Within-cluster within-period correlation
